# Phase 1 clinical trial of B-Cell Maturation Antigen (BCMA) NEX-T® Chimeric Antigen Receptor (CAR) T cell therapy CC-98633/BMS-986354 in participants with triple-class exposed multiple myeloma

**DOI:** 10.1038/s41375-025-02518-5

**Published:** 2025-02-05

**Authors:** Gayathri Ravi, Shambavi Richard, Shaji Kumar, Shebli Atrash, Michaela Liedtke, Gurbakhash Kaur, Benjamin Derman, P. Leif Bergsagel, Sham Mailankody, Philip McCarthy, Alok Shrestha, Lisa M. Kelly, Thomas Ly, Sharmila Das, Jerill Thorpe, Alison Maier, Divya Varun, Garnet Navarro, Michael R. Burgess, Kristen Hege, Ashley K. Koegel, Luciano J. Costa

**Affiliations:** 1 University of Alabama at Birmingham, Birmingham, AL USA; 2 Mount Sinai Health System, New York, NY USA; 3 Mayo Clinic, Rochester, MN USA; 4 Atrium Health Levine Cancer Institute, Charlotte, NC USA; 5 Stanford University, Stanford, CA USA; 6 UT Southwestern Medical Center, Dallas, TX USA; 7 University of Chicago, Chicago, IL USA; 8 Mayo Clinic, Scottsdale, AZ USA; 9 Memorial Sloan Kettering Cancer Center, New York, NY USA; 10 Roswell Park Comprehensive Cancer Center, Buffalo, NY USA; 11 wholly-owned subsidiaries of Bristol Myers Squibb Company, Princeton, NJ USA; 12 wholly-owned subsidiaries of Bristol Myers Squibb Company, Brisbane, CA USA; 13 wholly-owned subsidiaries of Bristol Myers Squibb Company, Seattle, WA USA

**Keywords:** Myeloma, Cancer immunotherapy

## Abstract

BCMA-targeted CAR T-cells transformed the treatment of relapsed and refractory multiple myeloma (RRMM), yet improvements are needed in manufacturing, toxicity and efficacy. We conducted a phase 1 clinical trial of BMS-986354, an autologous BCMA CAR T manufactured using an optimized NEX-T® process, in participants with triple-class exposed, RRMM. The 65 participants had a median of 5 (range 3–13) prior regimens, 39% had cytogenetic high-risk, 91% triple-class refractory, and 43% extra-medullar disease. Part A (dose-escalation) of the study enrolled participants in cohorts receiving 20 (*N* = 7), 40 (*N* = 24), or 80 (*N* = 11)x 10^6^ CAR + T-cells. In part B (expansion), an additional 23 participants were treated at the recommended phase 2 dose, 40 ×10^6^ CAR + T cells. Across dose levels, cytokine release syndrome (CRS) occurred in 82% (2% grade ≥3), neurotoxicity in 8% (2% grade ≥3), and infections in 32% of participants (5% grade ≥ 3). The response rate was 95%, with 46% achieving complete responses. Median progression-free survival was 12.3 months (95% CI 11.3–16). Compared to orvacabtagene autoleucel (same CAR construct, conventional manufacturing), BMS-986354 had higher proportion of T central memory cells, were less differentiated and had enhanced potency and proliferative capacity, supporting the use of NEX-T® in future CAR T development.

## Introduction

B cell maturation antigen (BCMA) or TNFRSF17 is a member of the tumor necrosis factor receptor superfamily with an important role in B cell survival and near ubiquitous expression in plasma cells, including in MM [1]. BCMA is also a relevant immunotherapy target in MM, validated by the therapeutic success of bispecific T cell engaging antibodies [2, 3], chimeric antigen receptor T cells (CAR T) [4, 5], and antibody-drug conjugates [6].

Autologous BCMA-directed CAR T cells have yielded very high rates of treatment response and durable disease control with the advantage of long treatment-free intervals. Two products, idecabtagene vicleucel (ide-cel) [4] and ciltacabtagene autoleucel (cilta-cel) [5], have obtained regulatory approval in some countries for patients with RRMM who have been exposed to prior therapies.

The existing commercial CAR T cell manufacturing processes have some important limitations. The manufacturing time of several weeks leads to the necessity of bridging therapy in many patients, with approximately 10% attrition between apheresis and infusion of cellular product. Cytokine release syndrome (CRS) is common and can be life-threatening in a small proportion of patients. Immune-effector cells associated neurotoxicity syndrome (ICANS) and non-ICANS neurotoxicity occur in < 20% but may cause short and/or long-term disability. Cytopenias are often prolonged and require intense management. Moreover, relapses appear unavoidable. There is therefore a need to optimize the manufacturing process and improve the safety and efficacy of autologous BCMA-directed CAR T cell therapies in order to expand access of these therapies and improve outcomes. CAR T therapy phenotypic attributes, such as enrichment for naïve early memory T cell subtypes, have been previously associated with improved clinical outcomes [7–9].

Here we report the results of a phase 1 clinical trial of BMS-986354, an autologous BCMA CAR T cell manufactured using the NEX-T® process, aiming to optimize phenotypic attributes, shorten manufacture time and yield myeloma control with favorable safety profile.

## Methods

BMS-986354 contains the same CAR construct present in orva-cel, with an anti-BCMA domain, a CD8 transmembrane domain, 4-1BB costimulatory domain and CD3-z signaling domain [10], but BMS-986354 is manufactured using the proprietary NEX-T® process. The manufacturing process involves selection of CD8+ and CD4 + T cells from the processed apheresis material followed by activation in cytokine enriched culture media. The activated T cells are transduced with BCMA lentivirus and are minimally expanded until harvest, followed by formulation and cryopreservation. The harvest duration leveraging shortened ex-vivo expansion for the NEX-T® process was determined by balancing robustness of the CAR expression and increased proportion of less differentiated T cells that demonstrated increased in vitro proliferative potential and cytolytic activity.

### Study design

This was a phase I, multi-center, open label study, performed in two parts consisting of dose escalation and dose expansion. The primary objectives were to evaluate safety and tolerability of increasing doses of BMS-986354 and obtain the recommended phase 2 dose (RP2D). The secondary and exploratory objectives were to evaluate safety, pharmacokinetic (PK) and preliminary efficacy at the RP2D dose based on overall response rate (ORR) and rates of CR/sCR according to the International Myeloma Working Group (IMWG) response criteria [11]. In addition, planned exploratory analysis also included and pharmacodynamic (PD) profile including immunophenotypic characterization of BMS-986354 clinical drug product performance.

In Part A, 3 dose levels (DL) of BMS-986354 were investigated: 20 (DL1), 40 (DL2) and 80 (DL3) x 10^6^ CAR + T cells. At each DL, the study intended to treat a minimum of 3 participants monitored for at least 28 days prior to adjudication of the safety profile of the respective DL. Dose escalation and de-escalation was determined by a Bayesian modified Toxicity Probability Interval 2 (mTPI-2) [12] algorithm with a target dose limiting toxicity (DLT) rate of 30% and an equivalence interval of 25% to 35%.

Participants were 18 years of age or older, had diagnosis of RRMM with confirmed progression during or within 12 months of last therapy or with no response and confirmed progression within the previous 6 months to most recent anti-myeloma treatment. Participants were required to have prior treatment with at least 3 distinct regimens, including a proteasome inhibitor (PI), an immunomodulatory agent (IMiD), an anti-CD38 monoclonal antibody and prior autologous stem cell transplantation (ASCT) unless ineligible. They were also required to have measurable disease with serum M-protein ≥0.5 g/dL and/or urine M-protein ≥200 mg/24-hour, or involved serum free light chain ≥ 10 mg/dL with abnormal kappa/lambda ratio. Additionally, participants had ECOG performance status of 0 or 1, absolute neutrophil count ≥  × 10^9^/L, platelet count ≥ 50 ×10^9^/L, creatinine clearance ≥ 60 ml/min and adequate cardiac and hepatic function among other inclusion criteria. The study excluded participants with known active or history of central nervous system involvement by MM, prior CAR T cell or another genetically modified T cell therapy, and prior therapy directed at BCMA.

Eligible participants underwent leukapheresis for collection of autologous T cells for product manufacturing. Participants received optional bridging therapy during the manufacturing period utilizing standard anti-myeloma drugs or combinations. Participants subsequently received lymphodepleting chemotherapy consisting of fludarabine 30 mg/m^2^/day and cyclophosphamide 300 mg/m^2^/day intravenously for 3 consecutive days, ending at least 2 (and a maximum of 9) days prior to infusion of BMS-986354. All participants were hospitalized for the first 14 days after BMS-986354 infusion and subsequently followed for 2 years for monitoring of toxicity and efficacy. After 2 years, participants were asked to participate in a long term follow up study (Study GC-LTFU-001).

Cytokine release syndrome (CRS) was graded according to criteria defined by Lee [13]. Other toxicities were graded utilizing the National Cancer Institute Common Terminology Criteria for Adverse Events (NCI-CTCAE) version 5.0.

### Ethics approval and consent to participate

The study was conducted in compliance with International Conference on Harmonization (ICH) and Good Clinical Practices (GCPs). *The protocol was approved by the respective Institutional Review Board or ethics committee at each of the 10 participating institutions (University of Alabama at Birmingham, Birmingham, AL; Mount Sinai Health System, New York, NY; Mayo Clinic, Rochester, MN; Mayo Clinic Scottsdale, AZ; Atrium Health Levine Cancer Institute, Charlotte, NC; UT Southwestern Medical Center, Dallas, TX; University of Chicago, Chicago, IL, Memorial Sloan Kettering Cancer Center, New York, NY; Roswell Park Comprehensive Cancer Center, Buffalo, NY; all in the USA)*. All subjects provided documented informed consent. An independent Data Safety Monitoring Board (DSMB) periodically reviews data to ensure safety, scientific validity and integrity of the study. The decision regarding dose escalation or de-escalation was made by a Safety Review Committee (SRC) composed by investigators in collaboration with the Sponsor. This trial is registered on ClinicalTrials.gov as study NCT04394650.

### Statistics

The study population consists of eligible participants infused with a conforming BMS-986354 product. Data are presented descriptively for each cohort in part A and for the expansion cohort in part B. Rates are described with 95% C.I. based on the exact binomial distribution. Secondary endpoints of duration of response (DOR), and PFS were analyzed utilizing the Kaplan-Meier method. The data cutoff for this analysis was June 16, 2023.

### Pharmacokinetics

The vector copy number (VCN) was determined by a validated Digital Droplet Polymerase Chain Reaction (ddPCR) method to detect lentivirus gag sequence in a matrix of human genomic DNA, isolated from whole blood. The preliminary summary results at data cutoff of June 16, 2023, by dose level are presented as transgene copies per microgram of genomic DNA. Exposure at data cutoff of August 7, 2022, was also assessed by an exploratory method known as flow cytometry. PK parameters derived from results by flow cytometry such as Area Under the Curve from 0 to 28 days AUC_0-28d_, Maximum Concentration (C_max_), and Time to Maximum Concentration (T_max_) were calculated using non-compartmental analysis (Phoenix WinNonlin 8.3).

### Drug product characterization

The memory phenotyping method is a flow based analytical method used to characterize the T cell memory composition and overall differentiation profile of the drug product by quantifying frequencies of bivariate populations including the following: CCR7 + CD45RA + , CCR7 + CD45RA-, CCR7-CD45RA-, and CCR7- CD45RA + .

The functionality of the drug product is assessed using the bulk cytokine release method, a multiplex assay that allows for the simultaneous measurement of multiple analytes in a single sample. The drug product is co-cultured overnight with the MM.1S cell line, which express BCMA endogenously. Upon incubation with the antigen-specific stimulated drug product, analytes/cytokines present will bind to their capture antibody and can be detected upon addition of a fluorescently labelled detection antibody. The reported concentration of each analyte in the test sample is determined relative to a standard curve generated with assay standards.

## Results

### Participants

**Fig. 1:**
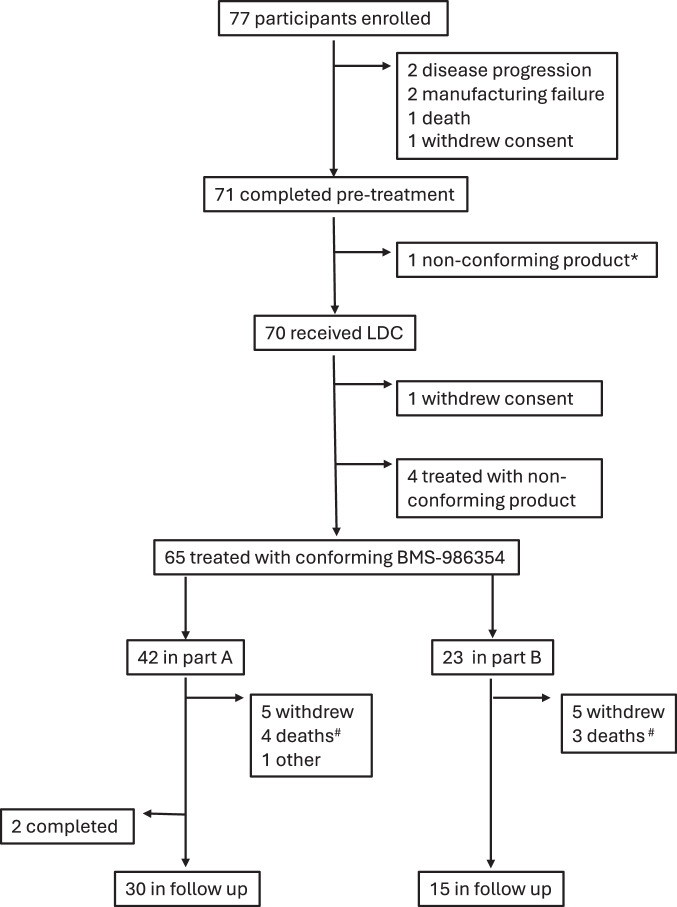
Consort diagram showing disposition of all participants enrolled in the study. *Participant chose to repeat leukapheresis and initiate new manufacturing. #Preceded by disease progression. LDC lymphodepleting chemotherapy.

There were 77 study participants enrolled between September 9, 2020, and August 24, 2022. Of those, 7 discontinued study participation after leukapheresis and before infusion of BMS-986354: 1 due to death, 2 due to disease progression, 1 due to withdrawal of consent, 1 due to failure to meet inclusion/exclusion criteria prior to lymphodepletion chemotherapy and 2 due to product manufacturing failure. One additional participant withdrew consent after initiating lymphodepleting chemotherapy and did not receive BMS-986354. Sixty-five participants (42 in dose escalation and 23 in dose expansion) were treated with conforming product and were the population of interest for safety and efficacy analyses (Fig. 1). In Part A, 7 participants were treated in DL1 (20 × 10^6^ CAR + T cells), 24 in DL2 (40 × 10^6^ CAR + T cells) and 11 in DL3 (80 × 10^6^ CAR + T cells). Five participants had non-conforming products, of those 1 repeated apheresis and manufacturing and eventually received a conforming product. All 4 remaining participants received non-conforming products, 1 in DL2 and 3 in DL3. Most common reason for non-conformity was insufficient number of CAR T-cells for the respective dose level (yet meeting or exceeding prior dose level with acceptable safety and efficacy).

Characteristics of participants are displayed in Table 1. Participants enrolled after a median of 6.3 (0.7–24.6) years from diagnosis and 5 (3–13) prior myeloma regimens. Median age at treatment was 63 (43-75) years, 25 (39%) had myeloma harboring high-risk chromosome abnormalities (17p13 deletion, 17p del, t4;14, or t14;16), and 28 (43%) had extramedullary plasmacytomas. Fifty-nine (91%) participants had triple-class (PI, IMiD and anti-CD38 monoclonal antibody) refractory MM and 31 (48%) had penta-drug (lenalidomide, pomalidomide, bortezomib, carfilzomib and daratumumab) refractory MM. Thirty-eight (59%) of participants were treated with optional bridging therapy between leukapheresis and lymphodepletion.

**Table 1:** Patient, disease, and prior treatment characteristics.

	Part A - Dose escalation			Part B- Dose Expansion	RP2D (Parts A + B)	Total
	DL1	DL2	DL3	DL2	DL2	
	N=7	N=24	N=11	N=23	N=47	N=65
Sex						
Male	6 (86%)	15 (63%)	5 (46%)	17 (68%)	32 (68%)	43 (66%)
Female	1 (14%)	9 (38%)	6 (55%)	6 (26%)	15 (32%)	22 (34%)
Age (in years)						
Median (range)	57 (45–71)	65.5 (46–75)	61 (47–73)	63 (43–74)	64 (43–75)	63 (43–75)
Age ≥ 65 years	1 (14%)	14 (58%)	5 (46%)	9 (39%)	23 (49%)	29 (45%)
Race						
White	5 (71%)	17 (71%)	9 (82%)	17 (74%)	34 (72%)	48 (74%)
Black or African Ameirican	1 (14%)	6 (25%)	2 (18%)	5 (22%)	11 (23%)	14 (22%)
Other/unknown	1 (14%)	1 (14%)	0 (0%)	1 (4%)	2 (4%)	3 (5%)
Hispanic Ethnicity	0 (0%)	2 (8%)	0 (0%)	3 (13%)	5 (11%)	5 (8%)
ECOG						
0	5 (71%)	10 (42%)	6 (55%)	12 (52%)	22 (47%)	33 (51%)
1	2 (29%)	14 (58%)	5 (46%)	11 (48%)	25 (53%)	32 (49%)
Median years from initial diagnosis (range)	7.2 (2.6–19.7)	5.1 (2.1–15.6)	7.3(1.8–24.6)	6.7 (0.7–14.6)	5.2 (0.7–15.6)	6.3 (0.7–24.6)
ISS at baseline						
1	1 (14%)	16 (67%)	6 (55%)	15 (65%)	31 (66%)	38 (59%)
2	3 (43%)	4 (17%)	3 (27%)	6 (26%)	10 (21%)	16 (25%)
3	3 (43%)	3 (13%)	2 (18%)	2 (9%)	5 (11%)	10 (15.4%)
Unavailable	0 (0%)	1 (4%)	0 (0%)	0 (0%)	1 (2%)	1 (2%)
R-ISS at baseline						
1	0 (0%)	10 (42%)	4 (36%)	6 (26%)	16 (34%)	20 (31%)
2	2 (29%)	6 (25%)	3 (27%)	9 (39%)	15 (32%)	20 (31%)
3	2 (29%)	2 (8%)	1 (9%)	2 (9%)	4 (9%)	7 (11%)
Unavailable	3 (43%)	6 (25%)	3 (27%)	6 (26%)	12 (26%)	18 (28%)
Cytogenetic abnormality						
del(17p)	3 (43%)	4 (17%)	3 (27%)	11 (48%)	15 (32%)	21 (32%)
t(4;14)	1 (14%)	2 (8%)	1 (9%)	3 (13%)	5 (11%)	7 (11%)
t(14;16)	0 (0%)	1 (4%)	1 (9%)	2 (9%)	3 (6%)	4 (6%)
Any high risk^a^	3 (43%)	6 (25%)	3 (27%)	13 (57%)	19 (40%)	25 (39%)
amp(1q)	2 (29%)	7 (29%)	1 (9%)	7 (30%)	1 4 (30%)	17 (26%)
Unavailable	1 (14%)	1 (4%)	0 (0%)	0 (0%)	1 (2%)	2 (3%)
Extramedullary plasmacytoma	4 (57%)	11 (46%)	3 (27%)	10 (44%)	21 (45%)	28 (43%)
LDH hgher than ULN	2 (29%)	4 (17%)	4 (36%)	6 (26%)	10 (21%)	16 (25%)
Prior ASCT	7 (100%)	23 (96%)	10 (91%)	19 (83%)	42 (89%)	59 (91%)
Median prior anti-myeloma regimens (range)	5 (4–8)	4 (3–13)	5 (3–9)	5 (3–7)	5 (3–13)	5 (3–13)
Prior Exposure						
Lenalidomide	7 (100%)	24 (100%)	11 (100%)	23 (100%)	47 (100%)	65 (100%)
Pomalidomide	7 (100%)	21 (88%)	11 (100%)	19 (83%)	40 (85%)	58 (89%)
Bortezomib	6(86%)	24 (100%)	10 (91%)	23 (100%)	47 (100%)	63 (97%)
Ixazomib	4 (57%)	8 (33%)	6 (55%)	5 (22%)	13 (28%)	23 (35%)
Carfilzomib	7 (100%)	22 (92%)	7 (64%)	20 (87%)	42 (89%)	56 (86%)
Daratumumab	7 (100%)	24 (100%)	11 (100%)	23 (100%)	47 (100%)	65 (100%)
Isatuximab	1 (14%)	0 (0%)	1 (9%)	1 (4%)	1 (2%)	3 (5%)
Bridging Therapy	4 (57%)	11 (46%)	6 (55%)	17 (74%)	28 (60%)	38 (59%)
Treatment refractorinesss						
Triple-class	7 (100%)	21 (88%)	9 (82%)	22 (96%)	43 (92%)	59 (91%)
Penta-drug	5 (71%)	12 (50%)	4 (36%)	10 (44%)	22 (47%)	31 (48%)
Last regimen	7 (100%)	24 (100%)	11 (100%)	20 (87%)	44 (94%)	62 (95%)

^a^del(17p), t(4;14), or t(14;16), *ISS* international staging system, *R-ISS* revised international staging system, *ASCT* autologous stem cell transplantation, *LDH* lactic dehydrogenase, *ULN* upper limit of normal, *ECOG* ECOG performance status.

### Safety

During part A, none of the 7 participants treated in DL1 experienced a DLT, 4 of 24 (16.7%) participants in DL2 (prolonged neutropenia and thrombocytopenia [*n* = 1], prolonged neutropenia [*n* = 2], decreased fibrinogen [*n* = 1]), and 3 of 11 (27.3%) participants in DL3 developed a DLT (prolonged neutropenia [*n* = 2], prolonged thrombocytopenia [*n* = 1]). All 3 dose levels were declared tolerable per pre-specified criteria. The SRC, considering safety, efficacy, PK and PD data, chose DL2 (40 × 10^6^ CAR + T cells) as the RP2D to expand in Part B.

All participants developed at least one treatment-emergent adverse event, irrespective of attribution. Fifty-three (82%) participants developed CRS, all grade 1 or 2 except for a single episode of grade 4 CRS in a participant in Part B. Median time between BMS-986354 infusion and onset of CRS was 4 (1–8) days, and median duration of CRS was 4 (1–9) days. There was no clear change in incidence, time of onset, duration, or severity of CRS with higher doses of BMS-986354 (Table 2). Treatments for CRS included tocilizumab in 48 (74%), dexamethasone in 31 (48%) and anakinra in 10 (15%) participants. Five (8%) participants experienced neurological toxicity events consisting with ICANS, 4 were grade 1, and 1 was grade 4. Median time of onset for neurotoxicity/ICANS was 5 (range 5–9) days and episodes lasted a median of 3 (range 1–12) days, requiring treatment with glucocorticoids in 3 (5%) participants. None of the participants developed cranial nerve palsy or Parkinsons-like symptoms (Table 3).

**Table 2:** Immune effector-mediated toxicities of special interest.

	Part A - Dose Escalation			Part B- Dose Expansion	RP2D (Parts A + B)	Total
	DL1	DL2	DL3	DL2	DL2	
	N=7	N=24	N=11	N=23	N=47	N=65
CRS						
Any grade	5 (71%)	20 (83%)	9 (82%)	19 (83%)	39 (83%)	53 (82%)
Grade 1	2 (29%)	17 (71%)	9 (82%)	14 (61%)	31 (66%)	42 (65%)
Grade 2	3 (43%)	2 (13%)	0 (0%)	4 (17%)	7 (15%)	10 (15%)
Grade 3	0 (0%)	0 (0%)	0 (0%)	0 (0%)	0 (0%)	0 (0%)
Grade 4	0 (0%)	0 (0%)	0 (0%)	1 (4%)	1 (2%)	1 (2%)
Grade 5	0 (0%)	0 (0%)	0 (0%)	0 (0%)	0 (0%)	0 (0%)
Median days from infusion to onset of CRS (range)	4 (1-8)	4 (2-6)	4 (2-6)	4 (2-6)	4 (2-6)	4 (1-8)
Median duration of CRS in days (range)	2 (2-9)	3 (1-6)	4 (2-8)	5 (2-7)	4 (1-7)	4 (1-9)
Treatment of CRS						
Tocilizumab	5 (71%)	18 (75%)	7 (64%)	18 (78%)	36 (77%)	48 (74%)
Dexamethasone	3 (43%)	12 (50%)	4 (36%)	12 (52%)	24 (51%)	31 (48%)
Anakinra	3 (43%)	3 (13%)	1 (9%)	3 (13%)	6 (13%)	10 (15%)
Neurotoxicity						
Any grade	1 (14%)	2 (8%)	1 (9%)	1 (4%)	3 (6%)	5 (8%)
Grade 1	1 (14%)	2 (8%)	1 (9%)	0 (0%)	2 (4%)	4 (6%)
Grade 2	0 (0%)	0 (0%)	0 (0%)	0 (0%)	0 (0%)	0 (0%)
Grade 3	0 (0%)	0 (0%)	0 (0%)	0 (0%)	0 (0%)	0 (0%)
Grade 4	0 (0%)	0 (0%)	0 (0%)	1 (4%)	1 (2%)	1 (2%)
Grade 5	0 (0%)	0 (0%)	0 (0%)	0 (0%)	0 (0%)	0 (0%)
Median days from infusion to onset of neurotoxicity (range)	8 (8–8)	7 (5–9)	5 (5–5)	5 (5–5)	5 (5–9)	5 (5–9)
Median duration of neurotoxicity in days (range)	2 (2–2)	3 (3–3)	2 (2–2)	11 (1–12)	3 (1–12)	3 (1–12)
Corticosteroids for treatment of neurotoxicity	1 (14%)	0 (0%)	0 (0%)	2 (9%)	2 (4%)	3 (5%)

*DL* dose level, *CRS* cytokine release syndrome, *RP2D* recommended phase 2 dose.

**Table 3:** Treatment-emergent adverse events occuring in 20% or more of participants.

	Part A - Dose Escalation						Part B- Dose Expansion		RP2D (Parts A + B)		Total	
	DL1 (*N* = 7)		DL2 (*N* = 24)		DL3 (*N* = 11)		DL2 (*N* = 23)		DL2 (*N* = 47)		(*N* = 65)	
	All grades	Gr 3-4	All grades	Gr 3-4	All grades	Gr 3-4	All grades	Gr 3-4	All grades	Gr 3-4	All grades	Gr 3-4
Any	7 (100%)	5 (71%)	24 (100%)	20 (83%)	11 (100%)	10 (91%)	23 (100%)	20 (87%)	47 (100%)	40 (85%)	65 (100%)	55 (85%)
Any hematologic	5 (71%)	5 (71%)	21 (88%)	20 (83%)	10 (91%)	10 (91%)	21 (91%)	20 (87%)	42 (89%)	40 (85%)	57 (88%)	55 (85%)
Neutropenia	4 (57%)	4 (57%)	19 (79%)	17 (71%)	10 (91%)	10 (91%)	17 (74%)	17 (74%)	36 (77%)	34 (72%)	50 (77%)	48 (74%)
Thrombocytopenia	2 (29%)	2 (29%)	10 (42%)	7 (29%)	7 (64%)	6 (55%)	13 (57%)	10 (44%)	23 (49%)	17 (36%)	32 (49%)	25 (39%)
Anemia	2 (29%)	2 (29%)	10 (42%)	8 (33%)	6 (55%)	5 (46%)	12 (52%)	9 (39%)	22 (47%)	17 (36%)	30 (46%)	25 (39%)
Lymphopenia	1 (14%)	1 (14%)	5 (21%)	5 (21%)	4 (36%)	4 (36%)	4 (17%)	3 (13%)	9 (19%)	8 (17%)	14 (22%)	13 (20%)
Infections	0 (0%)	0 (0%)	8 (33%)	2 (8%)	4 (36%)	0 (0%)	9 (39%)	1 (4%)	17 (36%)	3 (6%)	21 (32%)	3 (5%)
Fatigue	1 (14%)	0 (0%)	7 (29%)	0 (0%)	5 (46%)	0 (0%)	6 (26%)	0 (0%)	13 (28%)	0 (0%)	19 (29%)	0 (0%)
Diarrhea	2 (29%)	0 (0%)	5 (21%)	0 (0%)	6 (55%)	0 (0%)	5 (22%)	1 (4%)	10 (21%)	1 (2%)	18 (28%)	1 (2%)
Nausea	0 (0%)	0 (0%)	5 (21%)	0 (0%)	6 (55%)	0 (0%)	7 (30%)	0 (0%)	12 (26%)	0 (0%)	18 (28%)	0 (0%)
Constipation	0 (0%)	0 (0%)	5 (21%)	0 (0%)	2 (18%)	0 (0%)	7 (30%)	0 (0%)	12 (26%)	0 (0%)	14 (22%)	0 (0%)
Headache	1 (14%)	0 (0%)	6 (25%)	0 (0%)	3 (27%)	0 (0%)	6 (26%)	0 (0%)	12 (26%)	0 (0%)	16 (25%)	0 (0%)
Peripheral edema	0 (0%)	0 (0%)	6 (25%)	0 (0%)	3 (27%)	0 (0%)	4 (17%)	0 (0%)	10 (21%)	0 (0%)	13 (20%)	0 (0%)
Hypocalcemia	0 (0%)	0 (0%)	7 (29%)	0 (0%)	4 (36%)	0 (0%)	4 (17%)	0 (0%)	11 (23%)	0 (0%)	15 (23%)	0 (0%)
Hypokalemia	1 (14%)	0 (0%)	3 (13%)	0 (0%)	4 (36%)	0 (0%)	6 (26%)	0 (0%)	9 (19%)	0 (0%)	14 (22%)	0 (0%)
Hyponatremia	1 (14%)	0 (0%)	5 (21%)	0 (0%)	5 (46%)	0 (0%)	3 (13%)	0 (0%)	8 (17%)	0 (0%)	14 (22%)	0 (0%)
Alanine aminotransferase increase	0 (0%)	0 (0%)	4 (17%)	1 (4%)	4 (36%)	2 (18%)	5 (22%)	2 (9%)	9 (19%)	3 (6%)	13 (20%)	5 (8%)
Aspartate aminotransferase increase	1 (14%)	0 (0%)	4 (17%)	1 (4%)	4 (36%)	2 (18%)	4 (17%)	2 (9%)	8 (17%)	3 (6%)	13 (20%)	5 (8%)

*DL* dose level, *RP2D* Recommended Phase 2 Dose.

The most common treatment-emergent adverse events were hematologic, developing in 57 (88%) of participants, including 50 (76%) participants with neutropenia and 32 (49%) with thrombocytopenia. Grades 3/4 neutropenia and thrombocytopenia occurred in 48 (74%) and 25 (39%) participants respectively. Twenty-one participants (32%) developed infections, including 5 (7.7%) with grades 3/4 infections. Treatment-emergent adverse events occurring in at least 20% of participants are displayed in Table 3. There were no apparent differences in toxicity profile across different dose levels tested.

Two participants developed non-melanoma skin cancer (one basal cell, one squamous cell carcinoma). Three participants developed myelodysplastic syndrome (MDS). In the first case the patient had a newly demonstrated loss of chromosome 5q in the bone marrow sample obtained 1 month after BMS-986354. The patient developed progression of MM 12 months after treatment and received subsequent anti-MM therapy; subsequent analysis revealed expansion of the abnormal myeloid clone and additional cytogenetic abnormalities. In the second case, the patient developed MDS not otherwise specified 6 months after BMS-986354. There were no chromosome abnormalities and a next generation sequencing mutational panel revealed mutations in *TP53*; it is unknown if such mutations were present prior to BMS-986354. The third patient developed MDS 11 months after infusion of BMS-986354. Chromosome abnormalities identified included del(7q), del(5q). All 3 patient were alive at time of last follow up.

### Efficacy

**Fig. 2:**
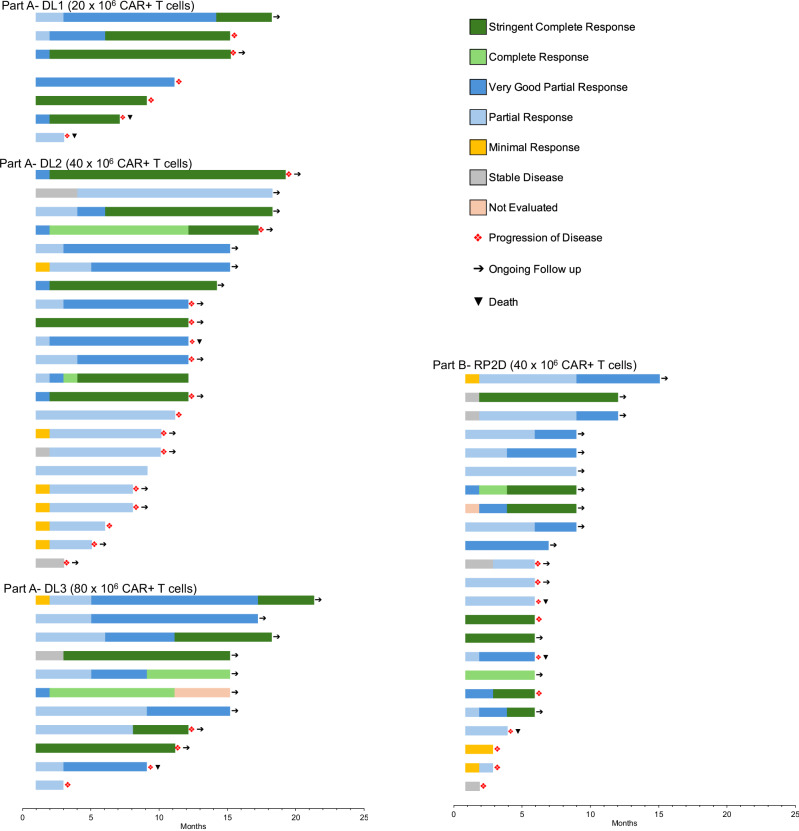
Depth and duration of response to BMS-986354. Swimmers’ plot displaying response trajectories for individual participants participants in each cohort.

**Fig. 3:**
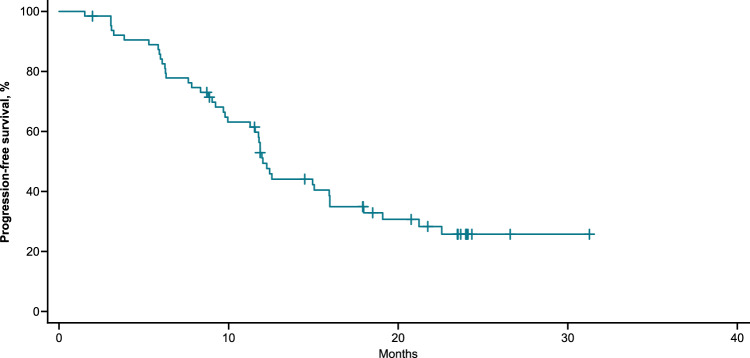
Progression-free survival of participants treated with BMS-98654. Kaplan-Meier curve displaying progression free survival for participants treated with BMS-98654 at the recommended phase 2 dose (RP2D).

Fast and deep responses were seen in all dose levels; 62 (95%, 95% C.I. 87%–99%) participants had partial response (PR) or better (PR, very good partial response, and CR/sCR), and 30 (46%, 95% C.I. 34%–59%) had CR/sCR. For the 47 participants treated at the RP2D, 44 (94%, 95% C.I. 82%–99%) had PR or better and 18 (38%, 95% C.I. 25%–54%) had CR/sCR (Table 4). Median time to response was 1.0 (range 0.9–4.1) month. Among the participants with PR or better, median duration of response was 11.3 (95% C.I. 10.6–17.1) months and 15.1 (95% C.I. 11.0-NE) months for participants who achieved CR/sCR (Fig. 2). Among the participants with PR or better, median follow time was 10.15 (1.0–18.7) months and 11.86 (4.6–18.7) months for participants who achieved CR/sCR. There were 25 participants with disease progression in Part A, and 10 in Part B. All (*n* = 5) deaths in Part A and Part B (*N* = 47) were preceded by disease progression. Median PFS was 12.3 months (95% C.I. 11.3–16.0) for all participants and 11.9 months (95% C.I. 9.2–18.0) for participants treated at the RP2D (Fig. 3).

**Table 4:** Anti-myeloma activity of BMS-986354.

	Part A - Dose Escalation			Part B- Dose Expansion	RP2D (Parts A + B)	Total
	DL1	DL2	DL3	DL2	DL2	
	*N* = 7	*N* = 24	*N* = 11	*N* = 23	*N* = 47	*N* = 65
Treatment best response (IMWG category)						
SD	0 (0%)	1 (4%)	0 (0%)	1 (4%)	2 (4%)	2 (3%)
MR	0 (0%)	0 (0%)	0 (0%)	1 (4%)	1 (2%)	1 (2%)
PR	1 (14%)	9 (38%)	1 (9%)	5 (22%)	14 (30%)	16 (25%)
VGPR	1 (14%)	4 (17%)	3 (27%)	8 (35%)	12 (26%)	16 (25%)
CR	0 (0%)	0 (0%)	2 (18%)	0 (0%)	0 (0%)	2 (3%)
sCR	5 (71%)	10 (42%)	5 (46%)	8 (35%)	18 (38%)	28 (43%)
≥CR	5 (71%)	10 (42%)	7 (64%)	8 (35%)	18 (38%)	30 (46%)
≥PR	7 (100%)	23 (96%)	11 (100%)	21 (91%)	44 (94%)	62 (95%)
Median time to response in mo. (range)	1.0 (0.9–1.0)	1.0 (0.9–4.1)	1.0 (0.9–2.9)	1.0 (1.0–3.1)	1.0 (0.9–4.1)	1.0 (0.9–4.1)
Median DOR for patients with ≥ PR in months (95% C.I.)	10.6 (2.1–15.1)	11.0 (8.3–17.1)	NE (10.3–NE)	NE (5.1–NE)	10.9 (8.2–17.1)	11.3 (10.6–17.1)
Median DOR for patients with ≥CR in months (95% C.I.)	15.0 (6.9–NE)	18.2 (13.8–NE)	NE (NE-NE)	NE (4.9–NE)	17.1 (10.8–NE)	15.1 (11.0–NE)
Median PFS in months (95% C.I.)	11.6 (3.1–16.0)	11.9 (9.7–18.0)	NE (11.3-NE)	NE (5.9–NE)	11.9 (9.2–18.0)	12.3 (11.3–16.0)

*DL* dose level, *IMWG* International Myeloma Working Group, *SD* stable disease, *MR* minimal response, *PR* partial response, *VGPR* very good partial response, *CR* complete response, *sCR* stringent complete response, *mo* months, *PFS* progression-free survival, *DOR* duration of response, *NE* not estimated.

### Product manufacturing and characteristics

**Fig. 4:**
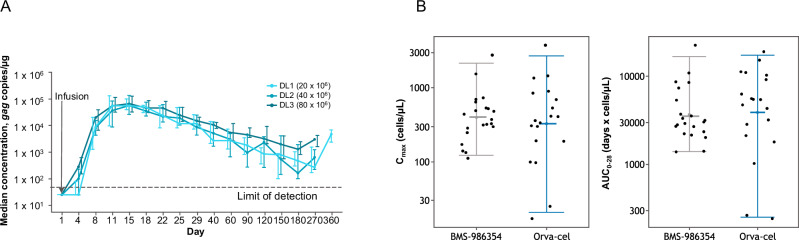
Pharmacokinetics of BMS-98654. BMS-986354 expansion and persistence at different dose levels as assessed by digital droplet polymerase chain reaction (**A**). Comparison of pharmacokinetic parameters between BMS-986354 at dose of 40 × 10^6^ CAR T cells and orva-cel at dose of 450 × 10^6^ CAR T cells (**B**), as assessed by flow cytometry. The error bars on panel A indicate interquartile range (IQR). The error bars on panel B indicate 95% CI of the mean. DL dose level, AUC0–28 Geometric mean area under the curve of the transgene level from time of dose to 28 days after infusion, Cmax geometric mean maximum blood concentration.

Orva-cel and BMS-98654 are transduced with the identical CAR construct, however, orva-cel is manufactured using a conventional, fully expanded process, whereas BMS-98654 is manufactured using an optimized NEX-T® process [10]. Multiple factors beyond manufacturing affect apheresis to infusion (“vein-to-vein”) in the context of a clinical trial, including post manufacturing quality control, staggering of subjects during dose-finding phase, recovery after bridging therapy, among others. Median time from apheresis to product availability was 30 days, median time from product availability to BMS-98654 infusion was 16 days and median time from apheresis to product infusion (“vein-to-vein”) was 45 days. Preliminary pharmacokinetic analysis using ddPCR showed robust in-vivo expansion at all dose levels (Fig. 4, panel A) with maximal expansion in approximately the first 2 weeks and persistence of detectable CAR T cells beyond 6 months after infusion. BMS-986354 at dose of 40 × 10^6^ CAR T cells produced similar C_max_ and similar AUC_0-28d_ derived using flow cytometry to orva-cel at approximately 10 times higher of a dose (450 × 10^6^ CAR T cells), demonstrating approximately tenfold increased proliferative capacity and potency on a cell-by-cell basis (Fig. 4, panel B).

**Fig. 5:**
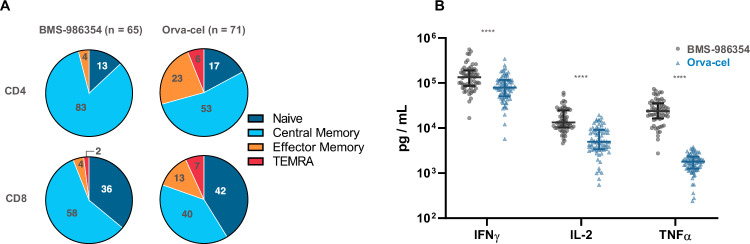
Phenotypic comparison of BMS-98654 and orva-cel. BMS-986354 exhibits a higher proportion of central memory and a lower proportion of effector memory and TEMRA in both CD4 and CD8 compartments (**A**). Following stimulation, BMS-98654 exhibits enhanced INFγ, IL-2 and TNFα when compared to orva-cel (**B**). Immune markers: naive-like, CCR7 + CD45RA + ; Central Memory CCR7 + CD45RA-; Effector Memory, CCR7-CD45RA- T effector memory RA (TEMRA), CD45RA + CCR7-. ****P ≤ 0.0001. IFNγ interferon gamma, IL-2 interleukin-2, TNFα tumor necrosis factor alpha.

Complete phenotypic characterization of 65 BMS-98654 products was compared with characteristics of 71 orva-cel products [10]. In the CD4 compartment, BMS-986354 cells had a higher proportion of a central memory phenotype (T_CM_, CCR7 + CD45RA-, 83% vs. 53%) and a lower proportion of effector memory phenotype (T_EM_, CCR7-CD45RA-, 4% vs 23%). Similarly, in the CD8 compartment 58% (vs. 40%) of cells were T_CM_, and 4% (vs. 13%) were T_EM_ (Fig. 5, panel A). When stimulated with MM.1S, which endogenously express BCMA, BMS-986354 secretes higher amounts of INFg, IL-2 and TNFa than orva-cel (Fig. 5, panel B).

## Discussion

BMS-986354 is a fully humanized anti-BCMA autologous CAR T cell therapy produced using an optimized NEX-T® process. In the present first-in-human phase 1 trial, we demonstrate a robust manufacturing process, reduced manufacturing time, improved phenotypic attributes with enrichment for central memory cells, and greater antigen-specific cytokine production when compared to orva-cel, despite the same CAR construct. An infusion of BMS-986354 leads to CAR T expansion and persistence similar to orva-cel when administered at a 10 times lower dose, demonstrating enhanced potency and proliferative capacity on a cell-by-cell basis. Persistence of whole blood concentration derived using ddPCR was up to 720 days (i.e. 24 months) post BMS-986354 dose at the highest nominal dose level of 80 million.

Long term disease control may be directly related to in vivo persistence of CAR T cells [14, 15]. In multiple studies, higher percentage of central memory T cells positively correlated with superior antitumor efficacy and better long-term disease control [14, 16]. The proliferative capacity of T cells is impacted by their state of differentiation. Stem cell-like memory T cells (T_SCM_), which are less differentiated, have increased potential to renew, replicate and generate all T cell subtypes including central memory (T_CM_) and effector memory (T_EM_) cells [17]. Unlike T_CM_ cells which can self-renew, T_EM_ are short lived and are responsible for early immune response [18] which correlates with potential for toxicity with adoptive immunotherapy and lacks the ability to sustain responses [18, 19]. CAR T cells undergo progressive differentiation after infusion, losing their proliferative capacity [20]. BMS-986354, with higher percentage of memory phenotype, offers a potentially efficacious CAR T manufacturing platform with a favorable safety profile. While low grade CRS was common, grade 3 or higher CRS was rare, seen only in 1 (2%) participant. For context, grade 3 CRS was seen in 5% of participants treated with ide-cel [4], 4% of participants treated with cilta-cel in the CARTITUDE-1 trial [5], and 35% of participants treated with cilta-cel in the CARTIFAN-1 trial [21]. Neurotoxicity occurred in 5 (8%) participants, all except for 1 were grade 1 and resolved in all. In contrast, neurotoxicity occurred in 18% of participants receiving ide-cel (including 3% grade 3) [4], 21% of participants receiving cilta-cel in CARTITUDE-1 (9% grade 3) [5]. None of the BMS-986354 recipients developed cranial nerve abnormalities or parkinsonism like neurologic complications. Infections were seen in 32% of participants, the majority were grades 1 or 2, and no deaths related to infection were noted. Cytopenias were common as anticipated (77% neutropenia and 49% thrombocytopenia) but prolonged neutropenia beyond 90 days was rare (5 participants, 8%). There were no deaths related to BMS-986354 toxicity. The maximal tolerated dose of BMS-986354 was not exceeded, and the study was expanded at the intermediate dose of 40 × 10^6^ cells. In aggregate, the safety of BMS-986354 compares favorably to available BCMA-directed CAR T cell therapies [4, 5, 21].

BMS-986354 had a high rate of responses (95%) including deep response (46% CR) in a cohort with a high percentage of patients with extramedullary disease (43%) and high-risk cytogenetic abnormalities (39%). Responses were comparable to orva-cel at higher doses with ORR of 91% and 40% CR rates [10]. Responses following BMS 986534 infusion were rapid, with median time to response of 1.0 month and sustained with median DOR of 11.3 months (95% C.I. 10.6–17.1). Increased depth of response correlated with durability of response, with median DOR of 15.1 months (95% C.I. 11.0-NE) for participants who achieved CR. There was no clear difference in depth or duration of response across the doses tested. The anti-myeloma activity of BMS-986354 compares favorably with historical results of conventional therapy in participants with triple-class exposed MM with response rates ~30% and median PFS < 5 months as shown in the observational MAMMOTH [22] and LOCOMMOTION [23] studies and in the control arm of the recent KARMMA-3 trial [24]. In fact, anti-myeloma activity compares favorably with other approved BCMA-directed therapy including CAR T cell [4] and bispecific T cell engagers [2, 3]. The overall response rate seen with BMS-986354 approaches what is reported with cilta-cel in the CARTITUDE-1 study in similar participant population, yet depth and duration of responses were numerically inferior [25].

The present study provides clinical validation that the NEX-T® process enables reliable manufacturing of an optimally expanded CAR T cell product with improved phenotypic characteristics, robust in-vivo expansion, and potent anti-myeloma effect when administered at low doses. BMS-986354 exhibited a tolerable safety profile, with only a single participant experiencing Grade 3 CRS or NT in a population with heavily pre-treated RRMM. While BMS-986354 is not currently being further developed, these results highlight the potential of the NEX-T® manufacturing process in future CAR T clinical development.

## Data Availability

The datasets generated during the current study are available from the corresponding author on reasonable requests for non-commercial use. Bristol Myers Squibb policy on data sharing may be found at https://www.bms.com/researchers-and-partners/clinical-trials-and-research/disclosure-commitment.html.
